# KIFC1 is essential for normal spermatogenesis and its depletion results in early germ cell apoptosis in the Kuruma shrimp, *Penaeus (Marsupenaeus) japonicus*

**DOI:** 10.18632/aging.102601

**Published:** 2019-12-29

**Authors:** Shuang-Li Hao, Wan-Xi Yang

**Affiliations:** 1 The Sperm Laboratory, College of Life Sciences, Zhejiang University, Hangzhou 310058, China

**Keywords:** spermatogenesis, apoptosis, KIFC1, acrosome formation, *Penaeus (Marsupenaeus) japonicus*

## Abstract

In order to explore the dynamic mechanisms during spermatogenesis of the penaeid prawns, the full length of *kifc1* was cloned from testis cDNA of *Penaeus japonicus* through RACE. Both semi-quantitative RT-PCR and Western blot results indicated that KIFC1 was extensive expressed in different tissue of *P. japonicus*. Compared with other tissue, the highest expression of KIFC1 occurred in the testis. According to the immunofluorescence results, the KIFC1 protein was detected at each stage of whole process of spermatogenesis. In the spermatogonial phase, KIFC1 mainly dispersed in cytoplasm and co-localized with microtubules, while abundant KIFC1 signal was detected in the nucleus of spermatocytes. At the early stage of spermatids, KIFC1 was transported from the nucleus into the cytoplasm, and it assisted microtubule assembly onto one side of the nucleus. Finally, in mature sperm, it was weakly expressed in the acrosome. This implies that KIFC1 may participate in the mitosis of spermatogonia, meiosis of spermatocyte, and acrosome formation during spermiogenesis; it may also play functions in acrosome maintaining in mature sperm. In addition, the results of KIFC1 knockdown by dsRNA injection *in vivo* reveal that decreased KIFC1 expression may induce aberrant microtubule assembly, and it leads to spermatogonia and spermatocyte apoptosis.

## INTRODUCTION

Mature and fertile sperm produced by spermatogenesis is the prerequisite for continuation of all gamogenetic species. Spermatogenesis is a complex and highly-ordered process that involves renewal and differentiation of spermatogonia stem cell, mitosis of spermatogonia, meiosis of spermatocyte, and the final dramatic morphological and cellular changes of spermiogenesis [1, 2]. For mammals, three prominent biological events occur during spermiogenesis, i.e., acrosome biogenesis, nucleus reshaping and tail formation. Finally, the swimming mature spermatozoa are produced [3]. However, spermatozoa of decapod crustaceans belong to non-flagella type and are immotile [4, 5]. The most notable morphological transformations during spermiogenesis in the testis are the formation of the special acrosomal complex, reshaping and decondensation of the nucleus [6–8]. The acrosome originates from small preacrosomal vesicles. The small preacrosomal vesicles are formed from endoplasmic reticula which coalesce into a large acrosomal vesicle and gradually adhere to the nucleus [9–12]. Meanwhile, the nuclei of sperm become more and more decondensed during this process [9]. The final spermatid in the testis is not fully developed and mature spermatozoa. When the spermatogenesis process in the testis is finished, sperm are transferred into the vas deferens and then are enwrapped in the spermatophores [13]. This is not only a spermatozoa transport process, but also a post-testicular sperm maturation process, called post-spermiogenesis [14].

Increasing studies suggest that the network of cytoskeleton and molecular motors is essential for the complicated dynamic spermatogenesis process [15, 16]. Microtubule is one of the major cytoskeletal components, which has a direct role in spermiogenesis in the form of manchette in mammal [17]. Although no manchette is reported by now in decapod crustaceans, microtubule bundles were observed [7]. Numerous papers have proven that cytoskeletal motors (kinesin, myosin and dynein) play essential roles during spermatogenesis such as the mitotic proliferation of spermatogonia [18–20], meiotic process of spermatocyte [21, 22], and spermiogenesis [17, 23]. Kinesin, a kind of microtubule-based motor protein, is also vital for different processes including microtubule organization [24] and vesicle transport [25]. The abnormal expression of kinesin motors results in spermatogenesis abnormities and even male infertility in various species. KIF3A motor protein is one of the kinesin II subunit, which acts as a key regulator of spermatogenesis. Depletion of KIF3A leads to serious damage in the formation of sperm tail and at the same time, it also affects the organization of manchette and the shaping of sperm head [23]. Another kinesin family member, KIF18A, which belongs to the kinesin-8 family, widely expresses in proliferative tissues and is an important regulator of chromosome alignment during mitosis [26]. Loss of KIF18A function in mouse germ cell results in chromosome alignment defects and undergoes mitotic arrest and apoptosis [22].

KIFC1 is a member of the kinesin-14 family, which moves along the microtubules from the plus end to the minus end. It has been identified in various species and has a critical function in vesicular transport [17, 27], Golgi apparatus positioning and structural integrity [28], microtubule organization [29], spindle assembly and chromosome segregation [30]. In addition, KIFC1 is reported with a critical role in centrosome clustering in various cancer cells such as hepatocellular carcinoma and it has been suggested as a potential prognostic biomarker and therapeutic target for cancer treatment [31]. Interestingly, KIFC1 is closely related to maintaining of the normal spermatogenesis of Mammalia, Reptile, Amphibia, Crustacea, Cephalopoda, and Sipunculida [17, 32–35].

The Kuruma shrimp *P. japonicus*, belongs to family Penaeidae, Crustacea [36]. It is one of the model animals for studying the non-flagella spermatogenesis in decapod crustacea. Up to date, the kinetics mechanism of spermatogenesis in *P. japonicus* remains unknown. The purpose of our research is to investigate how kinesin motors play function during penaeid spermatogenesis. Therefore, we cloned the full length cDNA of *kifc1* gene of *P. japonicus* and explored its function in penaeid prawn for the first time. The expression pattern during spermatogenesis and the result of KIFC1 knockdown by RNAi revealed KIFC1 may participate in mitosis and meiosis regulation, acrosome formation during spermatogenesis. The depletion of KIFC1 results in apoptosis of spermatogonia and spermatocytes.

## RESULTS

### The main features of *P. japonicus kifc1*

**Figure 1:**
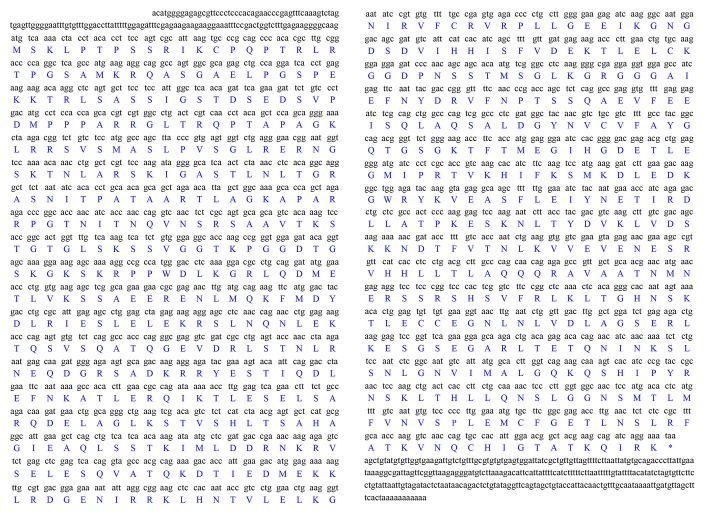
**Full-length cDNA of the *kifc1* from the testis of *P. japonicus*.** The corresponding amino acid sequence is shown below the nucleotide sequence. The full-length cDNA of this gene consists of a 136 bp 5’ untranslated region, a 297 bp 3’ untranslated region and a 2217 bp open reading frame which encodes 738 amino acids.

The full-length *kifc1* of *P. japonicus* is 2650 bp (GenBank accession number: MN072915). It contains 136 bp 5′ untranslated region (UTR), 2217 bp open reading frame (ORF), and 297 bp 3′ UTR. It encodes 738 amino acids and the predicted molecular weight is 81.10 kDa (Figure 1). The isoelectric point of this protein predicted by ExPASy-ProtParam tool is 9.47 (https://web.expasy.org/protparam/).

**Figure 2:**
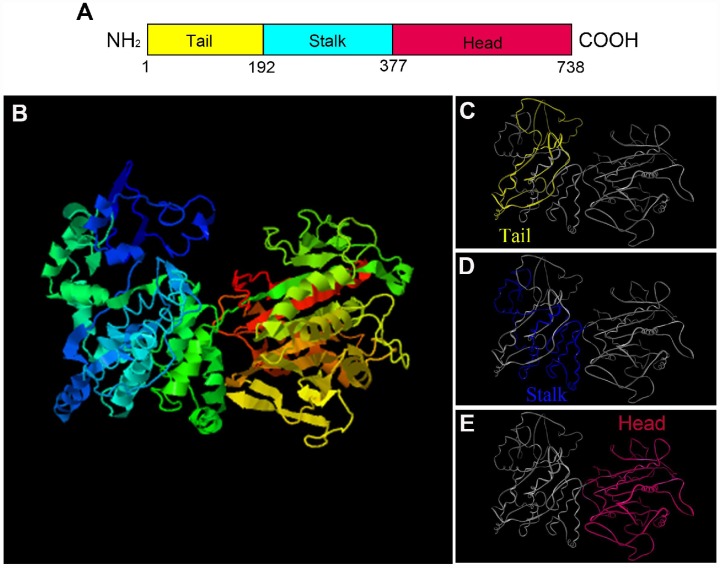
**The prediction of major structural features in *P. japonicus* KIFC1.** (**A**) Three structural domains of KIFC1 were shown in this figure. The motor domain labeled in red contains the conserved head which is from aa 377 to 738. The stalk region, also named coiled-coil domain, extends from aa 192 to 376 that labels in blue. The divergent tail domain labeled in yellow is from aa 1 to 191. (**B**) The putative 3-D structure of KIFC1 protein. (**C**) Tail domain (yellow part). (**D**) Coiled-coil domain (blue part). (**E**) Motor domain (red part).

The secondary structure prediction of *P. japonicus* KIFC1 showed three primary domains. A divergent tail at the amino terminal is from 1 to 192 amino acids, a coiled stalk begins from 193 to 377 amino acids, and a head at the carboxyl terminal is composed of the 378-738 amino acids, which contains the conserved motor domain and “walks” along the microtubule (Figure 2A). Additionally, we predicted the putative tertiary structure of KIFC1 protein, in which all three domains’ 3-D structures were observed clearly (Figure 2B–2E).

**Figure 3:**
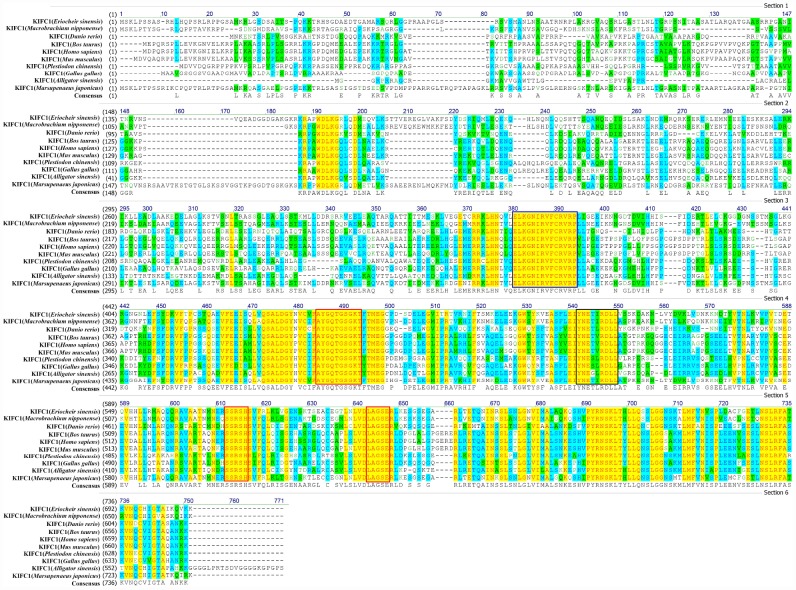
**Multiple sequence alignment of the KIFC1 protein in *P. japonicus* with that of other species.** The ELKGNIRVFCRVRP sequence (blue frame) is the KIFC conserved consensus. The AYGQTGSGKT, SSRSH, and LAGSE sequences (red frame) are the putative ATP binding sites. The YNETIRDLL sequence (black frame) is the microtubule-binding site.

**Figure 4:**
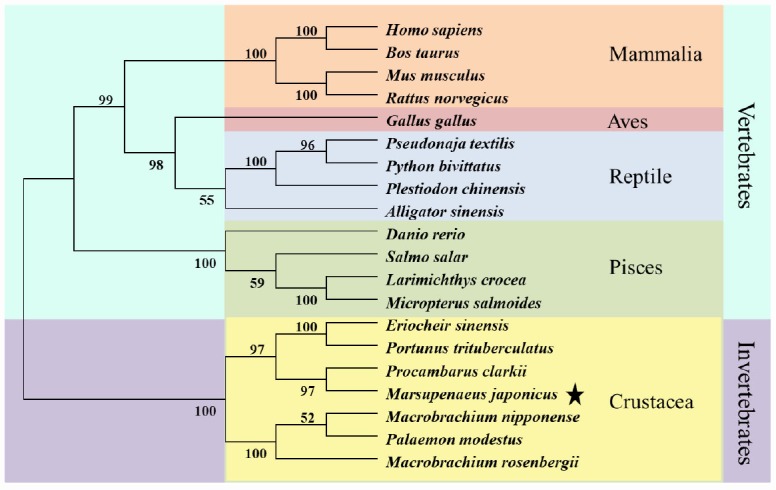
**Phylogenetic tree of KIFC1 protein from different species.** The phylogenetic tree was constructed through the neighbor-joining method with Mega 6 software. Mammlia, Aves, Reptile, Pisces, and Crustacea are included. The putative KIFC1 of *P. japonicus* constitutes a sister clade with it homologues of *Procambarus clarkia*.

We aligned KIFC1 of *P. japonicus* with its homologues of other species and found it has 66.1%, 57.7%, 35.5%, 36.1%, 35.8%, 36.6%, 35.5% and 33.1% identity with its homologues in *Eriocheir sinensis*, *Macrobrachium* *nipponensis*, *Danio rerio*, *Bos taurus*, *Homo sapiens*, *Mus musculus*, *Gallus gaullus*, and *Alligator sinensis*, respectively. The alignment result also showed a more conservative head region in KIFC1 when compared with the stalk and tail (Figure 3). In addition, there are three ATP binding sites (LAGSE, SSRSH and AYGQTGSGKT), a microtubule binding site (YNETIRDLL) and a KIFC conserved domain (ELKGNIRVFCRVRP) in the head region of *P. japonicus* KIFC1 (Figure 3). The phylogenetic analysis revealed the putative KIFC1 of *P. japonicus* constitutes a sister clade with it homologues of *Procambarus clarkii*, and also suggested it has a closest genetic relationship with *P. clarkii* among the examined species in this study (Figure 4).

### KIFC1 is highly expressed in reproductive system of *P. japonicas*

**Figure 5:**
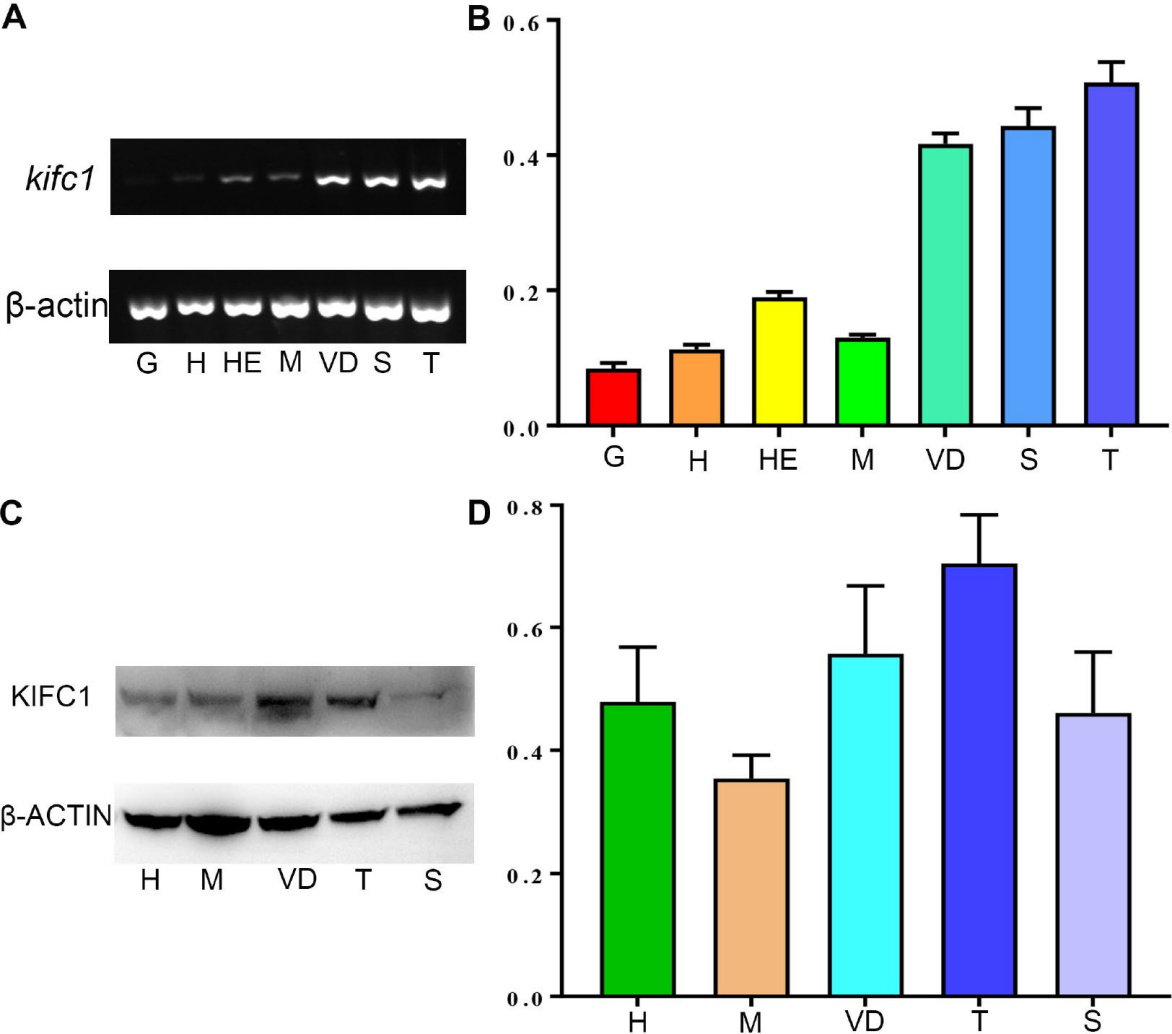
**The expression of KIFC1 in different tissues.** (**A**) *kifc1* mRNA expression in various *P. japonicus* tissues was shown by semi-quantitative RT-PCR analysis in the upper panel. The lower panel, β-actin, was used as a positive control. (**B**) A quantitative analysis of *kifc1* mRNA expression in various *P. japonicus* tissues was shown by Image J. The highest expression of *kifc1* appears in the testis. (**C**) The expression of KIFC1 protein in various *P. japonicus* tissues was shown in the upper panel by western blot. The lower panel, β-actin, was used as a positive control. (**D**) A quantitative analysis of KIFC1 expression in various *P. japonicus* tissues was shown by Image J. G: gill, H: heart, HE: hepatopancreas, M: muscle, S: spermatophore, T: testis, VD: vas deferens.

The expression of *kifc1* mRNA in different tissues of *P. japonicus* was determined using semi-quantitative RT-PCR. A 384 bp fragment of *kifc1* cDNA and a 179 bp fragment of *β-actin* were amplified in the heart, hepatopancreas, muscle, gill, vas deferens, spermatophore and testis (Figure 5A). *β-actin* was served as an internal control. Gray analysis by the Image J software indicated that *kifc1* mRNA was extensive expressed in all selected tissues, and the high expression occurred in the testis, vas deferens and spermatophore (Figure 5B). Meanwhile, we identified and analyzed KIFC1 protein expression in muscle, heart, testis, vas deferens and spermatophore by Western blots. A band about 81 kDa was recognized in all of these samples (Figure 5C). The expression trend of KIFC1 protein in all tissues is almost consistent with that of *kifc1* mRNA (Figure 5D), which implies that KIFC1 may have important roles in spermatogenesis of *P. japonicus*.

### KIFC1 participates in mitosis, meiosis as well as spermiogenesis of *P. japonicas*

**Figure 6:**
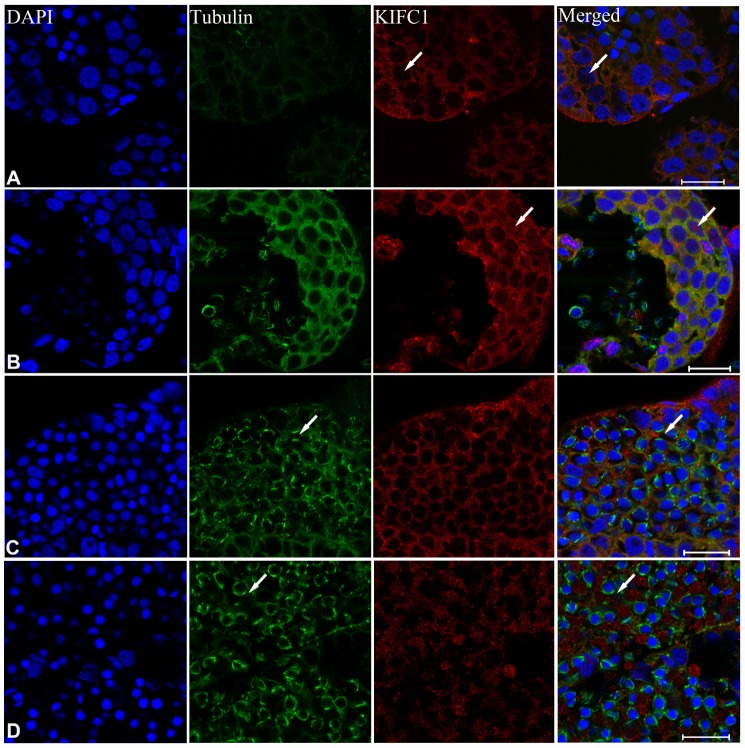
**Immunofluorescent localization of KIFC1 and tubulin during spermatogenesis in the testis of *P. japonicus*.** (**A**) Spermatogonia. KIFC1 and microtubules were co-localized in the cytoplasm. KIFC1 signal also can be detected in nucleus (white arrows). (**B**) Spermatocyte. The distribution of KIFC1 and microtubules in spermatocyte was same with that in spermatogonia. The difference was that both KIFC1 and microtubules signal in this stage were increased obviously (white arrows). (**C**) Early spermatid. KIFC1 and microtubules were distributed in the perinuclear cytoplasm. At this time, the microtubules started to assemble at one end (white arrows). (**D**) Late spermatid. A mass of KIFC1 spread over the spermatid cytoplasm. The microtubules exhibited spindle morphology around the sperm nucleus (white arrows). Blue: DAPI, Green: tubulin, Red: KIFC1. Scale bar= 20μm.

In order to explore the role of KIFC1 and microtubules during spermatogenesis of *P. japonicus*, we conducted the immunofluorescence further to examine the localization of them. In the spermatogonial phase, KIFC1 co-localized with tubulin in cytoplasm. Meanwhile, KIFC1 also dispersed in the nucleus (Figure 6A, arrows). In the spermatocyte phase, the signal of KIFC1 and tubulin in cytoplasm was obviously increased when compared with the spermatogonial phase. More importantly, a large number of KIFC1 signal was detected in the nucleus of spermatocyte (Figure 6B, arrows). In the early stage of spermatids, the microtubules began to assemble to one side of the nucleus (Figure 6C, arrows). The KIFC1 signal was exported from the nucleus, and spread over and co-localized with the microtubules in the cytoplasm (Figure 6C, arrows). In the late-stage spermatids, the microtubules exhibited characteristic spindle morphology around the nucleus (Figure 6D, arrows), while the signal of KIFC1 in this phase presented a relative increased (Figure 6D). In the control group, no signal of KIFC1 and tubulin was detected from the spermatogonia to late spermatids (Supplementary Figure 1). These results suggest that KIFC1 is likely to play essential roles during spermatogenesis of *P. japonicus* including mitosis of spermatogonia, meiosis of spermatocytes and the acrosome formation and maintaining.

**Figure 7:**
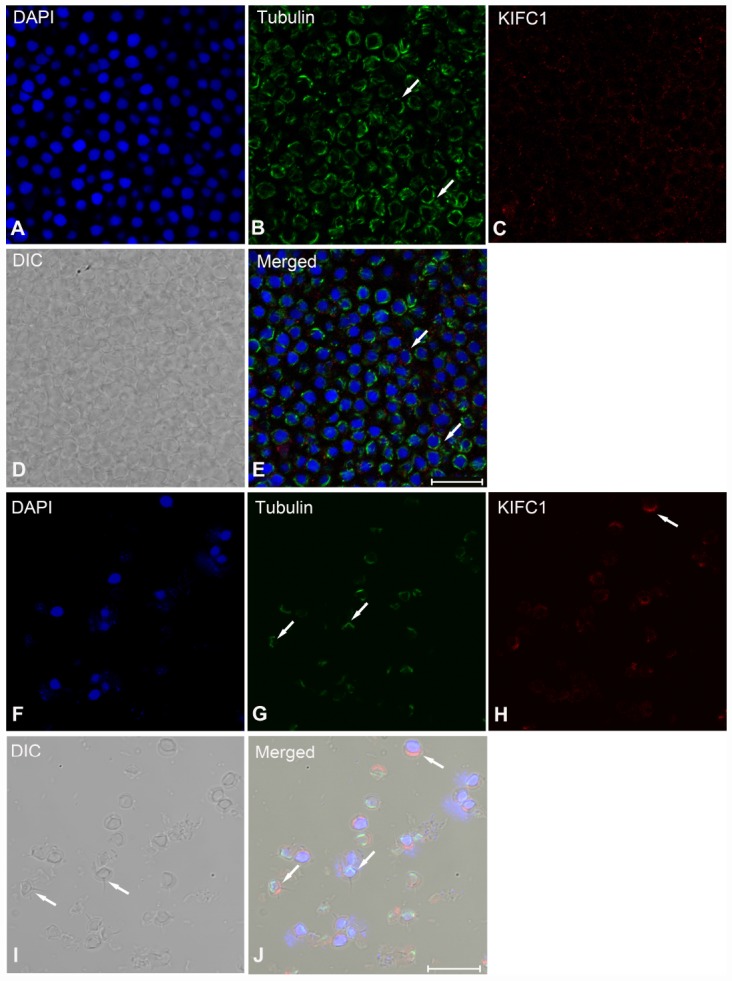
**Immunofluorescent localization of KIFC1 and tubulin during post-spermiogenesis in the vas deferens and in mature sperm from spermatophore of *P. japonicus*.** (**A**–**E**) Spermatid during post-spermiogenesis in the vas deferens. The signal of KIFC1 was very weak. The microtubules exhibited spindle morphology around the sperm nucleus (white arrows). (**F**–**J**) Mature sperm in spermatophore. KIFC1 and microtubules have completely separated localization in the mature sperm. From (**J**), it was clearly that KIFC1 was mainly localized at acrosome, while the microtubules distributed in the opposite side cytoplasm. Blue: DAPI, Green: tubulin, Red: KIFC1. Scale bar = 20μm.

During post-spermiogenesis in vas deferens (Figure 7), the microtubules also showed spindle-like structure around the nucleus (Figure 7B, arrows), while the signal of KIFC1 in this phase presented a relative reduction (Figure 7C). In mature sperm from the spermatophore, we can observe the nucleus, acrosome and spike under phase contrast microscopy (Figure 7I, arrows). The signal of microtubules and KIFC1 was completely not co-localized (Figure 7J, arrows). KIFC1 was localizes mainly in the acrosome, but the microtubules was close to the nucleus at the opposite end of acrosome (Figure 7G, 7H, 7J, arrows). These data show that KIFC1 has almost finished its task during spermatogenesis of *P. japonicus*, and continues to maintain the acrosome morphology in mature sperm in spermatophore.

### KIFC1 knockdown has a severely impact on microtubule distribution and results in early germ cell apoptosis in the testis of *P. japonicus*

**Figure 8:**
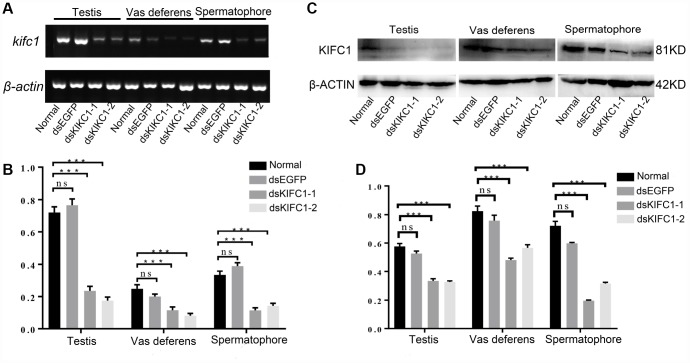
**Knockdown efficiency of *kifc1* by dsRNA injection *in vivo*.** (**A**) Expression level of *kifc1* mRNA was assayed by semi-quantitative RT-PCR analysis in the testis, vas deferens and spermatophore of *P. japonicus*. (**B**) A quantitative analysis of *kifc1* mRNA expression in the testis, vas deferens and spermatophore was determined by Image J. (**C**) Expression level of KIFC1 protein was detected by Western blot in the testis, vas deferens and spermatophore of *P. japonicus*. (**D**) A quantitative analysis of KIFC1 protein expression in the testis, vas deferens and spermatophore was determined by Image J. β-actin was used as an internal control.

In order to explore the function of KIFC1 during spermatogenesis of *P. japonicus*, we conducted the RNAi experiment through the injection of dsRNA to knockdown the expression of KIFC1 *in vivo*. The knockdown efficiency of *kifc1* was examined by semi-quantitative RT-PCR and Western blot (Figure 8A–8D). From the results of gray analysis, both dsKIFC1-1 and dsKIFC1-2 group had a significantly lower *kifc1* mRNA and KIFC1 protein expression than those of control groups (Figure 8B, 8D). Therefore, both of these two RNAi groups are feasible for further studies.

**Figure 9:**
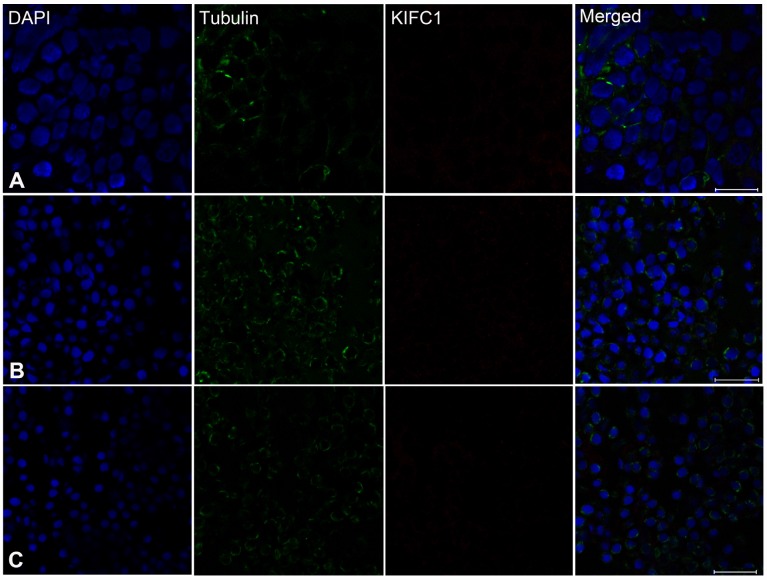
**Effects of *kifc1* knockdown on the distribution and assembly of the microtubules in the testis, vas deferens and spermatosphore.** (**A**) The microtubules in the testis. (**B**) The microtubules in vas deferens. (**C**) The microtubules in spermatosphore. When *kifc1* was knockdown, the microtubules were low expressed and abnormally assembled in spermatogonia and spermatocyte, while no obvious difference was found in spermatid from the vas deferens and mature sperm from spermatosphore. Blue: DAPI, Green: tubulin, Red: KIFC1. Scale bar = 20μm.

To determine whether there are effects on microtubule assembly, we observed the distribution of microtubules in the dsKIFC1-1 testis, vas deferens and spermatophore. In the dsKIFC1-1 testis, the signal of KIFC1 is hardly detectable. At the same time, the microtubule signal has an obvious decrease and it presents an abnormal accumulation in spermatogonia (Figure 9A, and Supplementary Figure 2A). However, in the dsKIFC1-1 vas deferens and spermatophore, although KIFC1 expression is knocked down, the expression and distribution of microtubules are notaffected (Figure 9B, 9C, and Supplementary Figure 2B, 2C). These results indicate that KIFC1 knockdown has a serious influence on the distribution of microtubules during the early spermatogenesis of *P. japonicus* including mitosis and meiosis, but not in the late stage, spermiogenesis.

**Figure 10:**
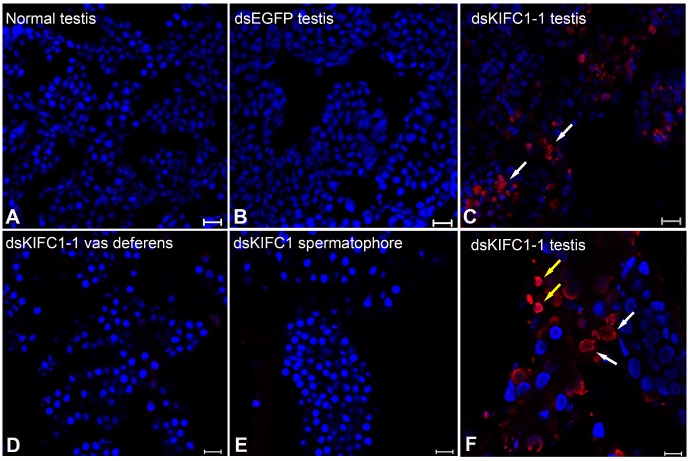
**Effects of *kifc1* knockdown on *P. japonicus* testis detected using the TUNEL assay.** (**A**) No apoptosis signal was detected in the normal testes. (**B**) No apoptosis signal was detected in dsEGFP testes. (**C**) Apoptosis signal was detected in dsKIFC1-1 testes. (**D**) No apoptosis signal was detected in dsKIFC1-1 vas deferens. (**E**) No apoptosis signal was detected in dsKIFC1-1 spermatophore. (**F**) In dsKIFC1-1 testes, apoptosis was taken place in spermatogonia (white arrows) and spermatocytes (yellow arrows). Blue: DAPI, Red: TUNEL. Scale bar = 20μm in **A**, **B** and **C**. Scale bar = 10μm in **D**, **E** and **F**.

In order to examine the effect of KIFC1 knockdown on spermatogenesis in *P. japonicus*, we conducted TUNEL assay. In the control groups (Normal and dsEGFP testis), no apoptosis is detected (Figure 10A, 10B). But the different results occur in the RNAi groups. Plenty apoptosis is detected in dsKIFC1-1 testes (Figure 10C), and apoptosis is taken place in spermatogonia and spermatocytes at high magnification (Figure 10F). In addition, we also detected apoptosis in the RNAi vas deferens and spermatophore, but the result shows that KIFC1 knockdown has no effect on the spermatids in vas deferens and the mature sperm in spermatophore, which is identical with that of microtubule assembly (Figure 10D, 10E).

## DISCUSSION

Numerous studies have reported that KIFC1 participates in the process of spermatogenesis in both vertebrates and invertebrates and KIFC1 is essential for acrosomal biogenesis and nuclear reshaping [17, 33, 37, 38]. However, there is no study on functions of KIFC1 during spermatogenesis of the species of Penaeidae until nowadays. In this paper, we suggest that KIFC1 has a critical role in spermatogenesis of the penaeid prawns by the spatial and temporal distribution exploring and RNA interference *in vivo*. The results of the spatial and temporal distribution imply that KIFC1 may have a crucial function in mitosis, meiosis and acrosome biogenesis. Further study on the function of KIFC1 through RNA interference *in vivo* showed that KIFC1 knockdown induced spermatogonia and spermatocyte apoptosis, which gave a strong evidence for KIFC1 function in mitosis and meiosis during spermatogenesis of *P. japonicus*.

### KIFC1 depletion affects the distribution of microtubules and induces the early germ cell apoptosis during spermatogenesis of *P. japonicas*

KIFC1 transports various cargos, including membrane-bounded organelles, vesicles and interacting proteins, along the microtubule from the plus end to the minus end [30, 39]. Additionally, since the KIFC1 contains a conserved nuclear localization signal in the tail domain, it can be transferred into the nucleus and participates in the regulation of mitosis and meiosis [40–42].

The mitotic and meiosis spindles are molecular machines composed of a bipolar array of microtubules, which contributes to chromosome segregation via microtubule sliding, growth as well as microtubule shrinkage [42, 43]. KIFC1 participates in spindle assembly and modulates spindle length [44]. In many kinds of cancer cells, KIFC1 has been demonstrated playing an essential role in bipolar spindle formation by clustering the multiple poles and maintaining chromosomal stability [45, 46]. Therefore, KIFC1 is closely associated with the dynamic of microtubules during both mitotic and meiosis. In order to explore whether KIFC1 knockdown has an impact on the microtubule dynamic during spermatogenesis of *P. japonicus*, we conducted RNA interference by dsRNA injection *in vivo*. From our results, decreased expression of KIFC1 leads to low microtubule signal and abnormal accumulation just in the early stages of spermatogenesis in *P. japonicus* testis. We speculated that the main reason for this phenomenon may be that special morphology assembled by microtubules has been accomplished before spermatids were released into the vas deferens (Figure 9).

KIFC1 is essential for mitosis not only in normal cells, but also in cancer cells. The KIFC1 deficiency affects the normal mitosis, as well as damages cell growth, proliferation and survival. Wei and Yang [47] found that the KIFC1 knockout 293T cells are hard to heal from scratched mechanical damage and cell death occurs in the wound healing assay. In KIFC1-depleted IMR-90 cells, although no obvious immediate cell death was observed, 20% less cells were detected compared with control group. Interestingly, when knocked down both KIFC1 and Mad2 in IMR-90 cells, the cell apoptosis was triggered suggesting their combined effect during mitosis [45]. KIFC1 presents upregulated in various cancer cells and is related to cancer progression [31, 48, 49]. Besides, the overexpression of KIFC1 can inhibit docetaxel-mediated apoptosis in breast cancer cells and prostate cancer cells [49, 50]. Several studies have shown that KIFC1 is associated with an apoptosis pathway, and inhibition of KIFC1 suppresses cell proliferation, but also induces the apoptosis pathway in different cancer cells [31, 49, 51]. In our study, we determined that cell apoptosis commonly occurred in the spermatogonia of the KIFC1 knockdown testis, which suggested the key function of KIFC1 in mitosis of spermatogonia and KIFC1 depletion resulting in spermatogonia apoptosis in *P. japonicus* (Figure 10).

In meiotic germ cells, KIFC1 also has a role in the regulation of spindle morphology and length, and in chromosome alignment and segregation. KIFC1 inhibition in mouse oocytes leads to aberrant spindle assembly and finally generates meiosis arrest [52]. Ncd, a KIFC1 orthologue in *Drosophila*, is reported to form the microtubule nucleating center at the spindle pole body during meiosis in oogenesis [53, 54]. In addition, KIFC1 depletion in *Procambarus clarkii* causes a whole suppression of spermiogenesis, which is speculated for the abnormal early spermatids undergo apoptosis due to an unsuccessful meiosis, or as a result of incomplete structure in KIFC1 knockdown testis [55]. All these results suggested that *kifc1* ablation resulted in the normal meiosis interruption or finally induced apoptosis in germ cell genesis [54, 55]. In this study, we detected that in the spermatocyte phase, the signal of KIFC1 and tubulin was obviously increased in cytoplasm by immunofluorescence. Besides, abundant KIFC1 molecules were transported into the nucleus, which implied its importance for normal meiosis of male *P. japonicus* (Figure 6B, arrows). Further analysis of the function of KIFC1 by TUNEL assays showed that KIFC1 knockdown triggered cell apoptosis in spermatocyte phase (Figure 10), which gave the evidence for its important role in normal meiosis during spermatogenesis of *P. japonicus*. Since no available germ cell lines of crustacean, we can’t conduct the experiments *in vitro* and explain the effects more clearly. However, our results are still very important.

### KIFC1 functions in the acrosome formation and nuclear morphogenesis during spermiogenesis

The motor protein KIFC1 has been proved to function in the formation of acrosome and morphogenesis of the nucleus in different species from lower animals like invertebrates to mammalians (Table 1). It is related to the nuclear membrane and acrosome in round and elongating spermatids in mammal [56]. In rats, KIFC1 may combine with nuclear factors and contribute to the formation of spermatid acrosome, as well as the nuclear changes, which gives the direct association of a molecular motor with the acrosome biogenesis and nuclear morphogenesis [17]. Wang et al. [32] came up with the initial hypothesis that KIFC1 keep the conserved role during spermiogenesis of cephalopods: participating in acrosome formation and nucleo-morphogenesis. And then, they detected that *kifc1* mRNA signal was abundant in spermatid undergoing acrosome formation and dramatic elongation in *Octopus tankahkeei*. In addition, during nuclear elongation and condensation KIFC1 was completely co-localizated with the cephalopod counterpart of manchette. It suggested that cephalopod KIFC1 took part in nuclear morphogenesis with assisting of the manchette-like perinuclear microtubules to transport specific cargoes [57]. During the swimming crab spermiogenesis such as *Portunus trituberculatus* and *Eriocheir sinensis*, several studies also have provided a preliminary evidence for the essential roles of KIFC1 in the acrosome biogenesis and nuclear morphogenesis [33, 37, 58]. However, in the phylum Sipuncula *Phascolosoma esculenta*, there is something different. Combined the detailed observations of the dynamic distribution of motor protein KIFC1, microtubules and mitochondria during spermiogenesis with the conserved function of KIFC1 in cargo transport and microtubule organization, the researchers proposed that KIFC1 functions in nuclear reshaping and midpiece formation in the spermiogenesis of *P. esculenta* [35].

**Table 1:** The functions of the motor protein KIFC1 in spermatogenesis of various species.

**Species**	**Effect stage**	**Cell type**	**Function**	**Reference**
Rat	Spermiogenesis	Spermatid	Transport vehicles; Acrosome formation	[17]
*Xenopus laevis*	Mitosis	Spermatogonia	Spindle bipolarity	[19]
*Eumeces chinensis*	Spermiogenesis	Spermatid	Acrosome biogenesis, nucleus reshaping and tail formation related	[60]
*Gekko japonicus*	Spermiogenesis	Spermatid	Acrosome formation; nuclear reshaping	[34]
*Larimichthys crocea*	spermiogenesis	Spermatid	Nuclear reshaping; flagellum formation	[61]
*Drosophila*	Meiosis	Oocyte	Meiosis II spindle assembly	[53]
*Marsupenaeus japonicus*	Mitosis; meiosis; spermiogenesis	Spermatogonia; spermatocyte; spermatid	Participate in mitosis and meiosis; Regulate microtubule assembly; Acrosome biogenesis and maintaining	The present study
*Eriocheir sinensis*	Spermiogenesis	Spermatid	Acrosome biogenesis and its maintenance	[37, 39]
*Octopus tankahkeei*	Spermiogenesis	Spermatid	Cytological transformation; sperm nuclear morphogenesis	[32, 57]
*Macrobrachium nipponense*	Spermiogenesis	Spermatid	Acrosome biogenesis; cellular transformation	[38]
*Exopalaemon modestus*	Spermiogenesis	Spermatid	Acrosome formation; nuclear shaping	[59]
*Portunus trituberculatus*	Spermiogenesis	Spermatid	Acrosome biogenesis; nuclear shaping	[33]
*Procambarus clarkii*	Spermiogenesis	Spermatid	Acrosome formation; nuclear shaping	[55]
*Phascolosoma esculenta*	Spermiogenesis	Spermatid	Nuclear reshaping; midpiece formation	[35]

The Natantia species of Decapoda is divided into three tribes: Caridea, Penaeid and Stenopodidea. In Caridea, several studies have been reported about KIFC1 participating in the spermiogenesis. Acroframosome (AFS) is a new structure consisted of microtubules, which is identified from *M. nipponense*. The microtubule bundle of AFS started from middle stage spermatids and localized around the developing acrosome, which assembled in an everted umbrella [38]. KIFC1 was then notarized to transport cargoes such as Golgi vesicles, mitochondria and other cellular components that results in acrosome formation and nuclear shaping with the help of AFS during the spermiogenesis of *Exopalaemon modestus* [59]. During *P. clarkii* spermiogenesis, although the AFS was not reported, there is no effect on KIFC1 functions in acrosome formation and nuclear shaping [55]. In this study, the KIFC1 randomly dispersed in the cytoplasm, and co-localized with the microtubules in early stage spermatid of *P. japonicus*, which is similar with that of *P. clarkia*, *M. nipponense* and *E. modestus* [38, 55, 59]. In the late-stage spermatids, the microtubules exhibited characteristic spindle morphology around the nucleus, while the signal of KIFC1 in this phase presented a relative reduction. In mature sperm from the spermatophore, the nucleus, acrosome and spike were clearly observed by phase contrast microscopy. The signal of microtubules and KIFC1 was completely separated. KIFC1 was localized mainly in the acrosome, but the microtubules were close to the nucleus in the opposite end of acrosome. This is a novel phenomenon, which has not reported in other species. We proposed that since sperm stored in spermatophore were completely mature, and what they need to do is just waiting for fertilization, the motor protein KIFC1 and microtubules have finished their mission. Owing to the limit of the interference duration and survival time after injection which are the two main factors influenced the outcome of this study, it is hardly for us to observe the morphological abnormality and to obtain the direct evidence for more specific function of KIFC1 in the acrosome formation and nuclear morphogenesis.

## CONCLUSIONS

In this study, we concentrated on the KIFC1 function in spermatogenesis of *P. japonicus*, and tried to uncover the reproduction and regulation mechanism. The temporal and spatial expression patterns of KIFC1 protein during spermatogenesis indicates that KIFC1 is likely to participate in the mitosis of spermatogonia, meiosis of spermatocyte, acrosome formation during spermiogenesis and acrosome maintaining in mature sperm. Besides, the result of KIFC1 knockdown by dsRNA injection verifies that deficiency KIFC1 causes aberrant microtubule expression and distribution, and also results in spermatogonia and spermatocyte apoptosis in the testes. In summary, our results provide a strong evidence for the important roles of KIFC1 during *P. japonicus* spermatogenesis. Because it’s hard to generate gene knockout crustacean by now, we attempted to knock down KIFC1 by dsRNA injection to elucidate its function during spermatogenesis *in vivo*, and its feasibility was verified. We hope this method could help to solve more scientific issues in crustacean study and other field.

## MATERIALS AND METHODS

### Animals and sample preparation

The adult male Kuruma shrimp were purchased from the aquatic market of Luojia Village in Hangzhou city (Zhejiang, China). We quickly sacrificed these shrimps and dissected to collect the heart, hepatopancreas, muscle, gill, epididymis, spermatophore and testis on ice. The detached heart, hepatopancreas, muscle, gill were put into liquid nitrogen immediately and then preserved at −80°C for RNA and protein extraction. The detached tissues of epididymis, spermatophore and testis were saved in two parts. One part was stored in –80°C for RNA and protein extraction. The other part was fixed in 4% paraformaldehyde (PFA) in phosphate buffered saline (PBS, 0.1 M, pH 7.4) at 4°C overnight, then dehydrated in 20% sucrose solution in PBS for 8 h and embedded with O.C.T. for immunofluorescence. These samples were stored at −40°C.

### RNA extraction and reverse transcription

Total RNA of the heart, hepatopancreas, muscle, gill, epididymis, spermatophore and testis were extracted according to the manufacturer's instructions of Trizol reagent (Takara, Dalian, China). All samples were dissolved and homogenized within the Trizol reagent by a homogenizer. The homogenate was sequentially dealt with chloroform, isopropanol and 75% ethanol to make the RNA precipitated. Finally, we dissolved the precipitated RNA from each tissue in 100 μl DEPC-H_2_O and measured its concentration by micro-spectrophotometer (Nano-100, Allsheng). All total RNA samples were stored at −80°C.

The PrimeScript® RT reagent Kit (Takara) was used to reverse transcript the total RNA into cDNA. All samples were stored at −20°C for semi-quantitative PCR analysis of KIFC1 mRNA expression in different tissues. In addition, the testis cDNA was used for *kifc1* gene cloning. The Smart RACE cDNA Amplification Kit (CloneTech) and the 3′-Full RACE Core Set with PrimeScript™ RTase (Takara) were used for 5′ and 3′ rapid-amplification of cDNA ends (RACE) reverse transcription, respectively.

### Full-length of *kifc1* cloning

It was reported that kinesin protein KIFC1 is conserved in its C-terminal. Thus, we try to design two pairs of primers (Table 2) based on the conserved region of *kifc1* from *Eriocheir sinensis* by using the Primer Premier 5 software to get the conserved sequence of the shrimp *kifc1* in the testis. All the primers were synthesized by the Shanghai Sangon Biological Engineering Technology Company (China). A cDNA fragment of *kifc1* was obtained by Nested Touchdown PCR using a Mygene Series Peltier Thermal Cycler (Hangzhou, China). The program ran as follows: 94°C for 5 min, 10 cycles of the touchdown program (94°C for 30 s, 55°C for 30 s, 72°C for 1.5 min, followed by 0.5°C decrease of the annealing temperature per cycle), followed by 25 cycles (94°C for 30 s, 50°C for 30 s, and 72°C for 1.5 min), and 10 min at 72°C for the final extension. The PCR products were then determined and separated by agarose electrophoresis. SanPrep column DNA gel extraction kit (Sangon Biotech) was used to purify the desired bands. Eventually, the purified fragment was linked into PMD19-T-vector (Takara), propagated in *Escherichia coli DH5α* (Takara) and then sent to the Sangon Biological Engineering Technology Company for sequencing.

**Table 2:** Primers used in *kifc1* cloning, semi-quantitative RT-PCR, and recombinant plasmid construction for dsRNA synthesis.

**Primer**	**Primer sequence**	**Purpose**
F1	GCVYTNGAYGGYTAYAAYGTSTG	PCR
R1	TTNGAGTTDCCDCCVAGRGA	PCR
F2	TATGGNCARACHGGHTCDGG	PCR
R2	CCRGCSARRTCHACMARRTT	PCR
3'F1	GGCTCAAACTCACAGGGCACAA	3’ RACE
3'F2	TGTTCATCACCTCCTGACGCTT	3’ RACE
5'R1	GACATTGCCGAGATTGGACAGAGATTTG	5’ RACE
KIFC1-RTF1	TGTCAAAGTCATCTGTGGGAGG	Semi-quantitative RT-PCR
KIFC1-RTR1	TATCTGGCGTTCAAGTGTGGC	Semi-quantitative RT-PCR
actin-F1	AGCCTTCCTTCCTGGGTATGG	Semi-quantitative RT-PCR
actin-R1	AGGGAGCGAGGGCAGTGATT	Semi-quantitative RT-PCR
dsKIFC1-F1	GGACTAGTGGGCTGGAGATACAAGGTAGAGG	Plasmid construction
dsKIFC1-R1	CCGCTCGAGAAGGGGGACACATTGACAAACAT	Plasmid construction
dsKIFC1-F2	GGACTAGTCTGTCAAAGTCATCTGTGGGAGG	Plasmid construction
dsKIFC1-R2	CCGCTCGAGTATCTGGCGTTCAAGTGTGGC	Plasmid construction
dsGFP-F1	GGACTAGTCGACGTAAACGGCCACAAGTT	Plasmid construction
dsGFP-R1	CCGCTCGAGATGGGGGTGTTCTGCTGGTAG	Plasmid construction

Based on this determined sequence, we designed two forward primers (3'F1 and 3'F2) for 3′ RACE and one reverse primer (5'R1) for 5’ RACE using the Primer Premier 5 software. Then we performed the Nested Touchdown PCR with the reverse primers from the 3′-Full RACE Core Set with PrimeScript™ RTase to abtain the 3′ RACE, and with the forward primer from the Smart RACE cDNA Amplification Kit (CloneTech) to get the 5′ RACE. We ran the 3’ RACE programs as follows: 94°C for 5 min, 10 cycles of the touchdown program (94°C for 30 s, 62°C for 30 s, 72°C for 0.5 min, followed by 0.5°C decrease of the annealing temperature per cycle), followed by 25 cycles (94°C for 30 s, 57°C for 30 s, and 72°C for 0.5 min), and 10 min at 72°C for the final extension. The 5′ RACE programs was as follows: 94°C for 5 min, 10 cycles of the touchdown program (94°C for 30 s, 68°C for 30 s, 72°C for 0.5 min, followed by 0.5°C decrease of the annealing temperature per cycle), followed by 25 cycles (94°C for 30 s, 63°C for 30 s, and 72°C for 0.5 min), and 10 min at 72°C for the final extension. The aimed PCR products were purified, amplified and sequenced by Sangon Biological Engineering Technology Company as described above.

### Sequence analysis and phylogenetic analysis

The full-length KIFC1 cDNA sequence was checked and assembled by Seqman (DNASTAR, Inc.). The amino acid sequence was translated by the online ExPASy translate tool (http://web.expasy.org/translate/). Multiple sequence alignments were performed with Vector NTI10 (Invitrogen). The secondary structure of KIFC1 protein was predicted with an online tool PSIPRED Workbench (http://bioinf.cs.ucl.ac.uk/psipred/). The 3-D structure of KIFC1 was predicted with an online server I-TASSER (http://zhanglab.ccmb.med.umich.edu/I-TASSER). The phylogenetic tree, neighbor-joining (NJ) method, was constructed according to the deduced amino acid sequence using MEGA 6. The aa sequence of KIFC1 homologues used in this study was downloaded from NCBI. Their Genbank accession numbers were as follow: *Homo sapiens* (NP_002254.2), *Bos taurus* (NP_001095406.1), *Mus musculus* (NP_001182227.1), *Rattus norvegicus* (NP_001005878.1), *Gallus gallus* (NP_001075167.1), *Pseudonaja textilis* (XP_026580999.1), *Python bivittatus* (XP_007442391.1), *P. chinensis* (AFP33411.1), *Alligator sinensis* (XP_006032359.1), *Danio rerio* (NP_571281.2), *Salmo salar* (ABQ59663.1), *Larimichthys crocea* (ALP83460.1), *Micropterus salmoides* (AKS36882.1), *E. sinensis* (ADJ19048.1), *Portunus trituberculatus* (AKS36885.1), *P. clarkii* (AJF36162.1), M*. nipponense* (AFP33456.1), *Palaemon modestus* (AIN36847.1), and *M. rosenbergii* (AFO63546.1).

### Semi-quantitative PCR analysis of KIFC1 mRNA expression

A pair of primers (KIFC1-RTF1, KIFC1-RTR1) (Table 2) were designed for analyzing the expression of KIFC1 mRNA in different tissues: heart, hepatopancreas, muscle, gill, seminiferous duct, spermatophore and testis. A pair of primers (actin-F1, actin-R1) (Table 2) was used to amplify a *β-actin* cDNA fragment as the control. The PCR program was run as follows: initial incubation at 94°C for 5 min; 35 cycles in a normal program: 94°C for 5 s, 60°C for 10 s, and 72°C for 15 s; 72°C for 5 min for the final extension. The software Image J was used to analyze the expression of KIFC1 mRNA in different tissues.

### Western blot

All tissues were homogenized in RIPA Lysis Buffer (Beyotime) with 1% protease inhibitors. The total protein was separated by 10% SDS-PAGE gels and then transferred to PVDF membranes (Millipore) by 200 mA, 90 min. After 2 h blocked with 5% non-fat milk in PBST buffer (0.1% Tween-20 in PBS, pH 7.2-7.4), the membranes were incubated overnight in primary antibodies at 4 °C (KIFC1 rabbit polyclonal antibody (1:500) designed and prepared by our lab, anti-ACTB rabbit polyclonal antibody (1:2000, BBI)). We washed the membranes in PBST (three times, 15min/time). Then the membranes were incubated with secondary goat-anti-rabbit HRP-conjugated antibody (1:4000, Beyotime) for 1h. After washing three times with PBST, an enhanced chemiluminescent kit (Beyotime) was used to examine the protein blots by chemiluminescence imaging. β-actin was used as control.

### Immunofluorescence

The samples were taken out from -40°C and cut into 8 μm sections with Cryostat microtome (Thermo Scientific, HM525 NX). The sections were permeabilized with 0.25% Triton X-100 in PBS for 15 min. Then, 5% BSA in PBST buffer was used to block the sections for 1 h. After that, the sections were incubated with KIFC1 rabbit polyclonal antibody (1:100) overnight at 4 °C. The negative control sections were incubated in 5% BSA without primary antibody. They were washed in PBST for 45 min (3 times, 15 min/time). Subsequently, the sections were incubated with Secondary Alexa Fluor 555-conjugated donkey-anti-rabbit antibody (1:500, Beyotime) and anti-α-Tubulin-FITC mouse monoclonal antibody (1:100, Sigma) at room temperature for 1 h. The negative control sections were incubated with just Secondary Alexa Fluor 555-conjugated donkey-anti-rabbit antibody (1:500, Beyotime). After washed 3 times like before, the sections were incubated with DAPI (Beyotime) for 5 min to stain the nucleus. Finally, the sections were mounted with Antifade Mounting Medium (Beyotime) and observed immediately with a confocal laser scanning microscope (Carl Zeiss, CLSM 710).

### RNA interference (RNAi)

Two pairs of specific primers of *kifc1* and one pair of EGFP negative control primers (Table 2) with restriction sites SpeI/ XhoI were designed by the Primer Premier 5 software. We cloned the target sequences into L4440 plasmid with two T7 promoters. The recombinant plasmid was used as template to synthesize the dsRNA by T7 RNAi Transcription kit (Vazyme). We examined the quality and concentration of dsRNA by 1% agarose gel electrophoresis and a micro-spectrophotometer (Nano-100, Allsheng), respectively.

The active adult male shrimps were selected for the RNA interference experiment. All selected shrimps were divided into four groups (Normal group, dsEGFP group, dsKIFC1-1 group and dsKIFC1-2 group, 12 individuals in each group) and cultured under a stationary temperature of 18°C in separated boxes. After the shrimps were adapted to the environment of the laboratory for 1 week, *in vivo* injections of dsRNA were given. Each shrimp was injected with 20μg dsRNA in 20 μl RNase-free water every three days, and lasted for two weeks. There was no treatment in normal group.

### TUNEL assay

The One Step TUNEL Apoptosis Assay Kit (Beyotime) was used for TUNEL assays. The prepared sections were fixed in 4% PFA for 30 min, and then permeabilized in 0.5% Triton X-100 in PBS for 5 min. After washed in PBS for two times, they were incubated with TUNEL reaction mixture for 1 h at 37 °C away from light, washed for three times in PBS, then stained with DAPI (Beyotime) for 5 min. Finally, the sections were mounted with Antifade Mounting Medium (Beyotime) and observed immediately with a confocal laser scanning microscope (Carl Zeiss, CLSM 710).

### Statistical analysis

Statistical analyses were performed by the GraphPad Prism 7.0 software. The t-test was used to determine the difference between two groups. Statistical significance was presented as *p < 0.05; **p < 0.01; ***p < 0.001, and no significance (ns). We indicated the results as means ± SEM.

## Supplementary Material

Supplementary Figures
