# Supplementary material for: KIFC1 is essential for normal spermatogenesis and its depletion results in early germ cell apoptosis in the Kuruma shrimp, Penaeus (Marsupenaeus) japonicus
Source: Aging (Albany NY). 2019 Dec 29;11(24):12773–92. doi: 10.18632/aging.102601 (PMC6949060; doi:10.18632/aging.102601)
Supplement: Supplementary Figures [file aging-11-102601-s001..pdf]

SUPPLEMENTARY FIGURES

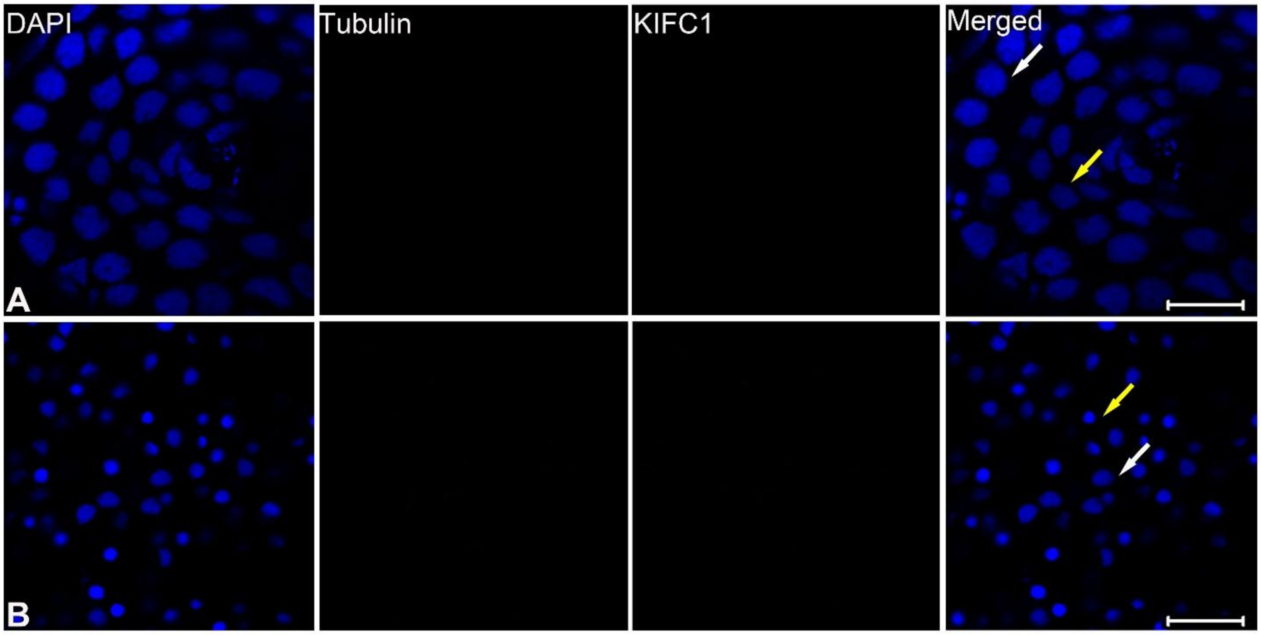

Supplementary Figure 1. Immunofluorescent localization of KIFC1 and tubulin during spermatogenesis in the control group of *P. japonicus* testis. (**A**) Spermatogonia and spermatocyte. No KIFC1 and microtubule signal was found in both spermatogonia (white arrow) and spermatocyte (yellow arrow). (**B**) Early spermatid and late spermatid. No KIFC1 and microtubule signal was found in both early spermatid (white arrow) and late spermatid (yellow arrow).Blue: DAPI, Green: tubulin, Red: KIFC1. Scale bar= 20µm.

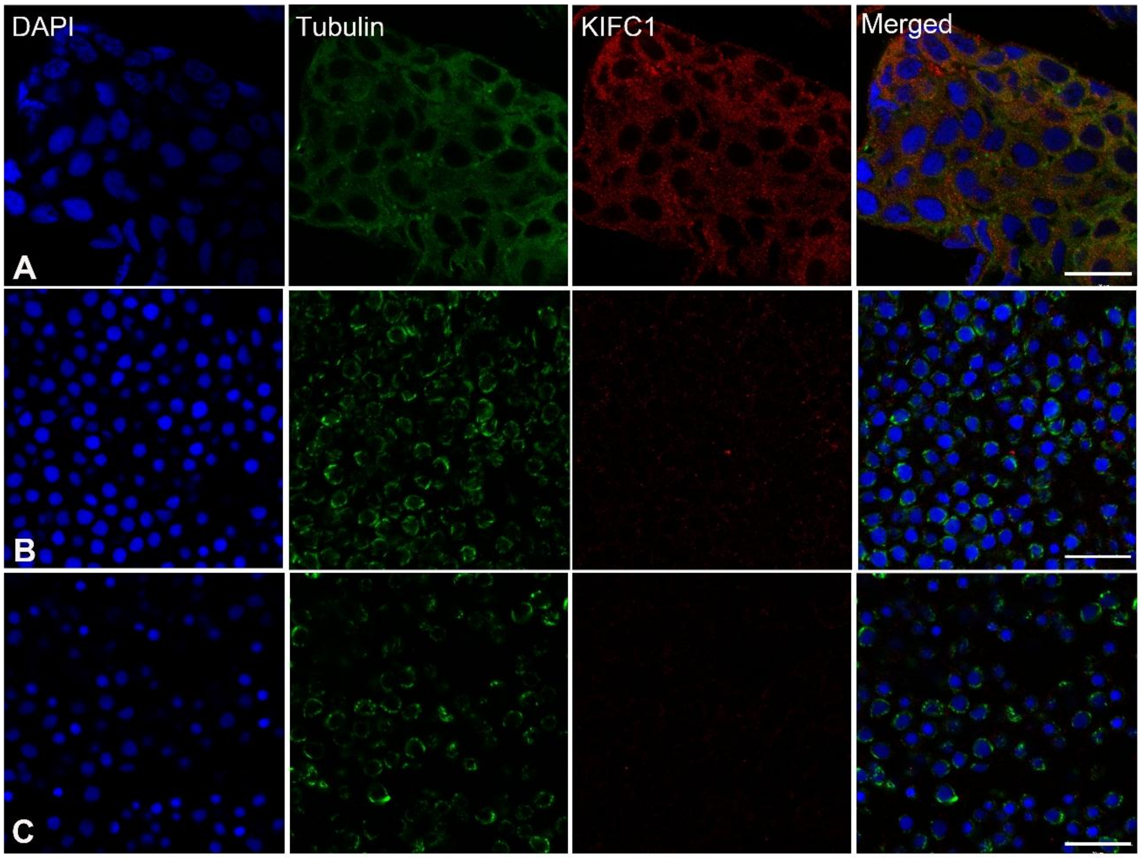

Supplementary Figure 2. Effects of the control dsRNA (dsEGFP) injected group on the expression and distribution of the microtubules in the testis, vas deferens and spermatosphore. (**A**) KIFC1 and microtubules in the testis. (**B**) KIFC1 and microtubules in vas deferens. (**C**) KIFC1 and microtubules in spermatosphore. In the control dsRNA (dsEGFP) injected group, the expression and distribution of KIFC1 and microtubules are normal. Blue: DAPI, Green: tubulin, Red: KIFC1. Scale bar = 20μm.
